# Endogenous GIP ameliorates impairment of insulin secretion in proglucagon-deficient mice under moderate beta cell damage induced by streptozotocin

**DOI:** 10.1007/s00125-016-3935-2

**Published:** 2016-04-06

**Authors:** Atsushi Iida, Yusuke Seino, Ayako Fukami, Ryuya Maekawa, Daisuke Yabe, Shinobu Shimizu, Keita Kinoshita, Yusuke Takagi, Takako Izumoto, Hidetada Ogata, Kota Ishikawa, Nobuaki Ozaki, Shin Tsunekawa, Yoji Hamada, Yutaka Oiso, Hiroshi Arima, Yoshitaka Hayashi

**Affiliations:** Department of Endocrinology and Diabetes, Nagoya University Graduate School of Medicine, 65 Tsurumai-cho, Showa-ku, Nagoya, 4668550 Japan; Department of Metabolic Medicine, Nagoya University Graduate School of Medicine, Nagoya, Japan; Yutaka Seino Distinguished Center for Diabetes Research, Kansai Electric Power Medical Research Institute, Kobe, Japan; Division of Molecular and Metabolic Medicine, Kobe University Graduate School of Medicine, Kobe, Japan; Research Center of Health, Physical Fitness and Sports, Research Institute of Environmental Medicine, Nagoya University, Nagoya, Japan; Department of Genetics, Division of Stress Adaptation and Recognition, Research Institute of Environmental Medicine, Nagoya University, Furo-cho, Chikusa-ku, Nagoya, 4648601 Japan; Department of Oral and Maxillofacial Surgery, Nagoya University Graduate School of Medicine, Nagoya, Japan

**Keywords:** Dipeptidyl peptidase IV, GIP, GLP-1, Glucagon, Hyperglycaemia, Insulin, Insulin secretion, Streptozotocin

## Abstract

**Aims/hypothesis:**

The action of incretin hormones including glucose-dependent insulinotropic polypeptide (GIP) and glucagon-like peptide-1 (GLP-1) is potentiated in animal models defective in glucagon action. It has been reported that such animal models maintain normoglycaemia under streptozotocin (STZ)-induced beta cell damage. However, the role of GIP in regulation of glucose metabolism under a combination of glucagon deficiency and STZ-induced beta cell damage has not been fully explored.

**Methods:**

In this study, we investigated glucose metabolism in mice deficient in proglucagon-derived peptides (PGDPs)—namely glucagon gene knockout (*Gcg*KO) mice—administered with STZ. Single high-dose STZ (200 mg/kg, hSTZ) or moderate-dose STZ for five consecutive days (50 mg/kg × 5, mSTZ) was administered to *Gcg*KO mice. The contribution of GIP to glucose metabolism in *Gcg*KO mice was also investigated by experiments employing dipeptidyl peptidase IV (DPP4) inhibitor (DPP4i) or *Gcg*–*Gipr* double knockout (DKO) mice.

**Results:**

*Gcg*KO mice developed severe diabetes by hSTZ administration despite the absence of glucagon. Administration of mSTZ decreased pancreatic insulin content to 18.8 ± 3.4 (%) in *Gcg*KO mice, but ad libitum-fed blood glucose levels did not significantly increase. Glucose-induced insulin secretion was marginally impaired in mSTZ-treated *Gcg*KO mice but was abolished in mSTZ-treated DKO mice. Although *Gcg*KO mice lack GLP-1, treatment with DPP4i potentiated glucose-induced insulin secretion and ameliorated glucose intolerance in mSTZ-treated *Gcg*KO mice, but did not increase beta cell area or significantly reduce apoptotic cells in islets.

**Conclusions/interpretation:**

These results indicate that GIP has the potential to ameliorate glucose intolerance even under STZ-induced beta cell damage by increasing insulin secretion rather than by promoting beta cell survival.

**Electronic supplementary material:**

The online version of this article (doi:10.1007/s00125-016-3935-2) contains peer-reviewed but unedited supplementary material, which is available to authorised users.

## Introduction

Glucagon is secreted from pancreatic alpha cells and contributes to promoting hepatic glucose production [1]. Diabetic patients show a paradoxical secretion of glucagon in response to meal test [2] and such diabetic hyperglucagonaemia is thought to be due to the relative deficiency of insulin action [3]. Thus, blockade of glucagon action is considered to be a novel target for glucose-lowering drug development [3].

Several animal models deficient in glucagon action have been reported, including prohormone convertase 2 knockout mice [4, 5], glucagon receptor knockout (*Gcgr*^−/−^) mice [6], mice treated with glucagon receptor antisense oligonucleotide [7] and mice having pancreas-specific *Arx* ablation [8]. All of these animal models show lower blood glucose levels, suggesting that glucagon plays a major role in hepatic glucose production and the maintenance of blood glucose levels. Moreover, several studies demonstrated that such animal models do not develop hyperglycaemia after beta cell destruction by streptozotocin (STZ) treatment [8–11], suggesting that glucagon plays an indispensable role in hyperglycaemia caused by beta cell destruction.

Glucose-dependent insulinotropic polypeptide (GIP) and glucagon-like peptide-1 (GLP-1) are incretins released from intestinal K- and L cells, respectively, and potentiate insulin secretion from beta cells in a glucose-dependent manner [12, 13]. GLP-1 is produced from proglucagon, which also serves as a precursor of glucagon. Several animal models deficient in glucagon action show markedly elevated plasma GLP-1 levels [5–7, 14], suggesting that GLP-1 might contribute to normoglycaemia under STZ-induced beta cell destruction via extra-pancreatic effects. We previously generated mice lacking proglucagon-derived peptides (PGDPs), including glucagon and GLP-1 (*Gcg*KO mice) [15]. *Gcg*KO mice display increased insulin sensitivity due to glucagon deficiency and enhanced early-phase insulin secretion in a GIP-dependent manner [16]. In the present study, we investigated glucose metabolism in *Gcg*KO mice administered with STZ. We found that *Gcg*KO mice developed marked hyperglycaemia under the severe insulin deficiency caused by STZ-induced beta cell destruction despite the absence of glucagon. However, *Gcg*KO mice displayed normoglycaemia under moderate insulin deficiency caused by moderate beta cell damage. We also investigated involvement of GIP in resistance to beta cell damage in *Gcg*KO mice.

## Methods

### Materials

Acetaminophen, STZ and BSA were obtained from Sigma-Aldrich (St Louis, MO, USA). Anagliptin, a dipeptidyl peptidase IV (DPP4) inhibitor (DPP4i) was provided from Sanwa Kagaku Kenkyusho Co. (Nagoya, Aichi, Japan).

### Animals

*Gcg*KO mice in a C57BL/6 background were established and maintained as previously reported (*Gcg*^*tm1Yhys*^) [15]. *Gcg*KO heterozygous and wild-type mice were used as controls. *Gcg*KO and heterozygous mice express green fluorescent protein (GFP) in cells expressing the glucagon gene. GIP receptor knockout (*Gipr*KO) mice, originally generated in the C57BL/6 background (*Gipr*^*tm1Yse*^) [17], were obtained from RIKEN BRC (Tsukuba, Japan) through the National Bio-Resource Project of the Ministry of Education, Culture, Sports, Science and Technology, Japan. *Gcg*KO and *Gipr*KO mice were intercrossed to obtain *Gcg*–*Gipr* double knockout (DKO) mice [16]. All mice used in this study were male. All procedures were conducted according to protocols and regulations approved by the Nagoya University Animal Experiment Committee.

### STZ treatment

To induce moderate damage to beta cells in the mice, STZ was administered i.p. at a dose of 50 mg (kg body weight [BW])^−1^ for five consecutive days (moderate-dose STZ [mSTZ]) [18]. To induce beta cell destruction, 200 mg STZ (kg BW)^−1^ was injected after 16 h-fast (high-dose STZ [hSTZ]) [19].

### DPP4i treatment

Anagliptin was administered through feed water at a concentration of 0.625 mg/ml from 7 days before starting STZ treatment to the end of the experiment.

### Glucose tolerance tests

OGTT and i.p. glucose tolerance test (IPGTT) (2 g/kg BW) were performed as previously described [16].

### Gastric emptying

Liquid and solid phase of gastric emptying were assessed as previously described [20].

### Biochemical analyses

Blood glucose levels were measured using an Antsense Blood Glucose Meter (Horiba, Kyoto, Japan). Plasma GIP and insulin were measured using Rat/Mouse GIP (total) ELISA (Merck Millipore, Darmstadt, Germany) and Mouse Insulin ELISA Kit (Morinaga Institute of Biological Science, Kanagawa, Japan) as previously reported [21]. Blood samples for measurements of biologically intact GLP-1 and GIP were collected using BD P800 tubes (Becton Dickinson, Franklin Lakes, NJ, USA). Plasma levels of biologically intact GLP-1 and GIP were evaluated using the following immunoassays according to manufacturers’ instructions: GLP-1, Active GLP-1 (ver. 2) Kit (Meso Scale Discovery, Rockville, MD, USA) and GIP, Mouse GIP, Active form Assay Kit (Immuno-Biological Laboratories, Gunma, Japan).

### Pancreatic insulin and GIP content analysis

Pancreatic tissue was homogenised in Krebs-Ringer buffer (pH 7.4) on ice. Tissue homogenate was extracted overnight in acid-ethanol (1.5% (vol./vol.) HCl in 75% (vol./vol.) EtOH). Tissue extracts were diluted 1:100 or 1:200 for insulin measurement. Diluted extracts were measured by HTRF Insulin Kit (Cisbio Bioassays, Codolet, France). Pancreatic insulin content was corrected by tissue weight for analysis. Tissue extracts were diluted 1:3 with Krebs-Ringer buffer for GIP measurement and protein content assay. Diluted extracts were measured by Rat/Mouse GIP (total) ELISA (Merck Millipore) and BCA protein assay (Sigma-Aldrich). Pancreatic GIP content was corrected by protein content for analysis.

### Immunohistochemistry

Tissue preparation and analyses have been described in detail previously [16]. Pancreas tissue was collected 1 day after the final administration of mSTZ to analyse cleaved caspase-3 and at 9 weeks of age to analyse beta cell mass. For analysis of cleaved caspase-3, sections were treated in citrate buffer-based Target Retrieval Solution (Dako, Glostrup, Denmark) before primary antibody incubation. Sections were incubated overnight with primary antibodies to cleaved caspase-3 (1:300; #9661; Cell Signaling Technology, Danvers, MA, USA) and/or insulin (1:150; ab7842; Abcam, Cambridge, MA, USA). Secondary antibodies (Alexa fluor 488, 594; 1:500; A-11073, A-11037 or A-11076; Molecular Probes, Eugene, OR, USA) were applied and incubated for 90 min at room temperature. Antibodies used are commercially available and widely used for immunohistochemistry. Fluorescent images were taken using BZ-9000 Fluorescence Microscope (Keyence, Osaka, Japan). The number of islets used to analyse cleaved caspase-3 was 355. The total areas of islets, insulin-positive cells (beta cells) and cleaved caspase-3 were analysed using BZ-X analyser software (Keyence).

### Statistical analysis

Results are presented as means ± SEM. Statistical significance was evaluated by ANOVA or a Student’s *t* test using GraphPad Prism 6 for Windows (GraphPad Software, La Jolla, CA, USA).

## Results

### Severe hyperglycaemia is induced by hSTZ-induced beta cell ablation in *Gcg*KO mice

**Fig. 1:**
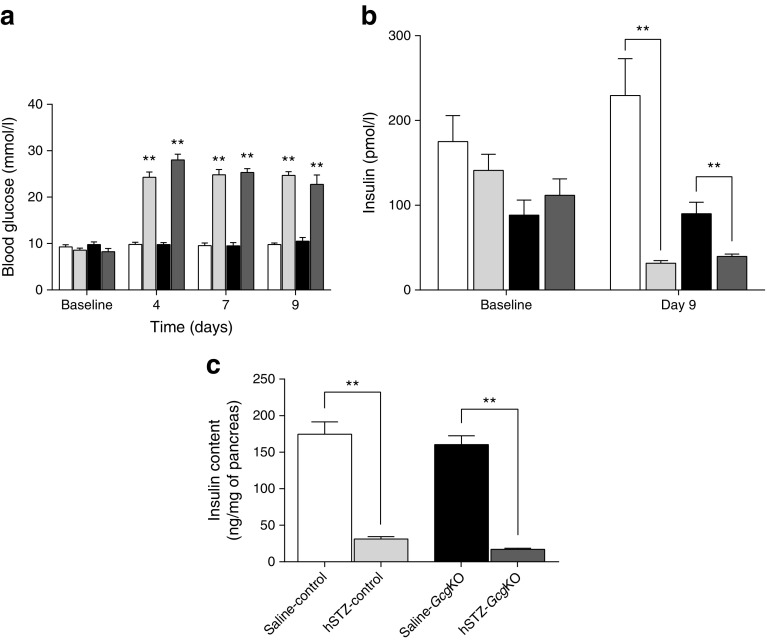
(**a**) Blood glucose levels, (**b**) plasma insulin levels and (**c**) pancreatic insulin content in control and *Gcg*KO mice after hSTZ treatment (200 mg/kg). STZ was i.p. injected at a dose of 200 mg/kg BW after overnight (16 h) fast. (**a**) Blood glucose levels under ad libitum-fed states were measured in both *Gcg*KO and control mice before (baseline) and at 4, 7 and 9 days after hSTZ administration. (**b**) Plasma insulin levels under ad libitum-fed states were measured before and 9 days after hSTZ injection. (**c**) Pancreatic insulin content was analysed at 9 days after hSTZ injection. **p < 0.01, saline-treated vs hSTZ-treated. White bars, saline-control; light grey bars, hSTZ-control; black bars, saline-*Gcg*KO; dark grey bars, hSTZ-*Gcg*KO. *n* = 6–9 per group

To evaluate glucose metabolism under beta cell dysfunction in the *Gcg*KO and control mice, STZ was given at a high dose (200 mg/kg, one shot; Fig. 1). As shown in Fig. 1a, both *Gcg*KO and control mice displayed severe hyperglycaemia at 4, 7 and 9 days after hSTZ administration. In accord with the blood glucose levels, plasma insulin levels were significantly decreased to <40 pmol/l in both groups (Fig. 1b). Pancreatic insulin content was analysed 9 days after hSTZ administration, and showed nearly 90% of the beta cells to be ablated in both *Gcg*KO and control mice (Fig. 1c). These findings demonstrate that severe destruction of beta cells causes hyperglycaemia even in *Gcg*KO mice, which lack PGDPs including glucagon, indicating that glucagon action is not requisite for persistent hyperglycaemia.

### Normoglycaemia is maintained in *Gcg*KO mice after mSTZ treatment

**Fig. 2:**
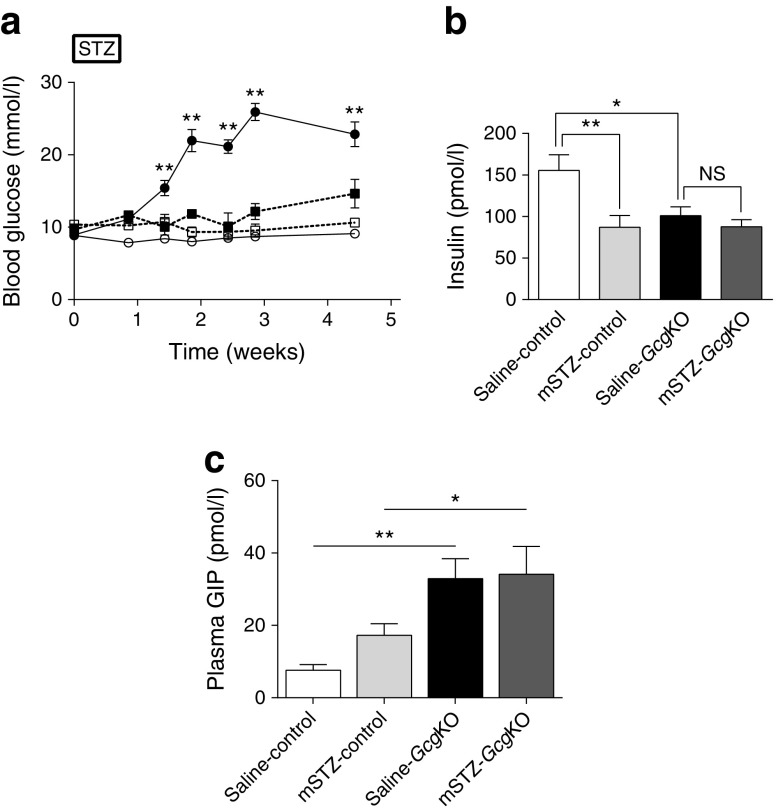
(**a**) Blood glucose levels, (**b**) plasma insulin levels and (**c**) plasma GIP levels in control and *Gcg*KO mice after mSTZ treatment in ad libitum-fed states. STZ or saline was i.p. injected once daily at a dose of 50 mg/kg BW for five consecutive days. *p < 0.05, **p < 0.01; NS, not significant. Significance of blood glucose levels is expressed vs saline-treated mice (**a**). Plasma insulin levels were evaluated at 5 weeks (**b**). Significance of plasma GIP levels was tested for control vs *Gcg*KO mice (**c**). White circles and solid line, saline-control; black circles and solid line, mSTZ-control; white squares and dashed line, saline-*Gcg*KO; black squares and dashed line, mSTZ-*Gcg*KO (**a**). White bars, saline-control; light grey bars, mSTZ-control; black bars, saline-*Gcg*KO; dark grey bars, mSTZ-*Gcg*KO (**b**, **c**). *n* = 6–12, saline-control; *n* = 12–15, mSTZ-control; *n* = 4–10, saline-*Gcg*KO; *n* = 5–12, mSTZ-*Gcg*KO

We next analysed glucose metabolism in mSTZ (50 mg/kg once daily for 5 days)-treated control and *Gcg*KO mice (Fig. 2). Beta cell damage in mice treated with mSTZ was milder than that in mice treated with hSTZ by analysis of the fluorescent images of cleaved caspase-3 (Electronic Supplementary Material [ESM] Fig. 1). In contrast to hSTZ administration, mSTZ treatment revealed differential effects on blood glucose levels in control and *Gcg*KO mice. As shown in Fig. 2a, blood glucose levels in mSTZ-treated *Gcg*KO (mSTZ-*Gcg*KO) mice were not significantly elevated compared with those in saline (154 mmol/l NaCl)-treated *Gcg*KO (saline-*Gcg*KO) mice, whereas those in mSTZ-treated control (mSTZ-control) mice were significantly elevated at 2 weeks and thereafter. After mSTZ administration, plasma insulin levels in control mice were significantly decreased, but they were not changed in *Gcg*KO mice (Fig. 2b). Plasma GIP levels were significantly higher in *Gcg*KO than in control mice. To determine whether absence of GLP-1 and increased GIP modify motility of the gastrointestinal tract in *Gcg*KO mice, the gastric emptying rate was evaluated to assess solid phase gastric emptying. Acetaminophen absorption test also was performed to assess liquid phase gastric emptying. There was no significant difference between control and *Gcg*KO mice in either liquid (152.02 ± 6.79 μmol/l in control; 161.66 ± 13.94 μmol/l in *Gcg*KO; *p* = 0.557) or solid phase (39.3 ± 11.3% in control; 28.5 ± 12.2% in *Gcg*KO; *p* = 0.539) gastric emptying rate (ESM Fig. 2).

### GIP contributes to glucose homeostasis in *Gcg*KO mice

**Fig. 3:**
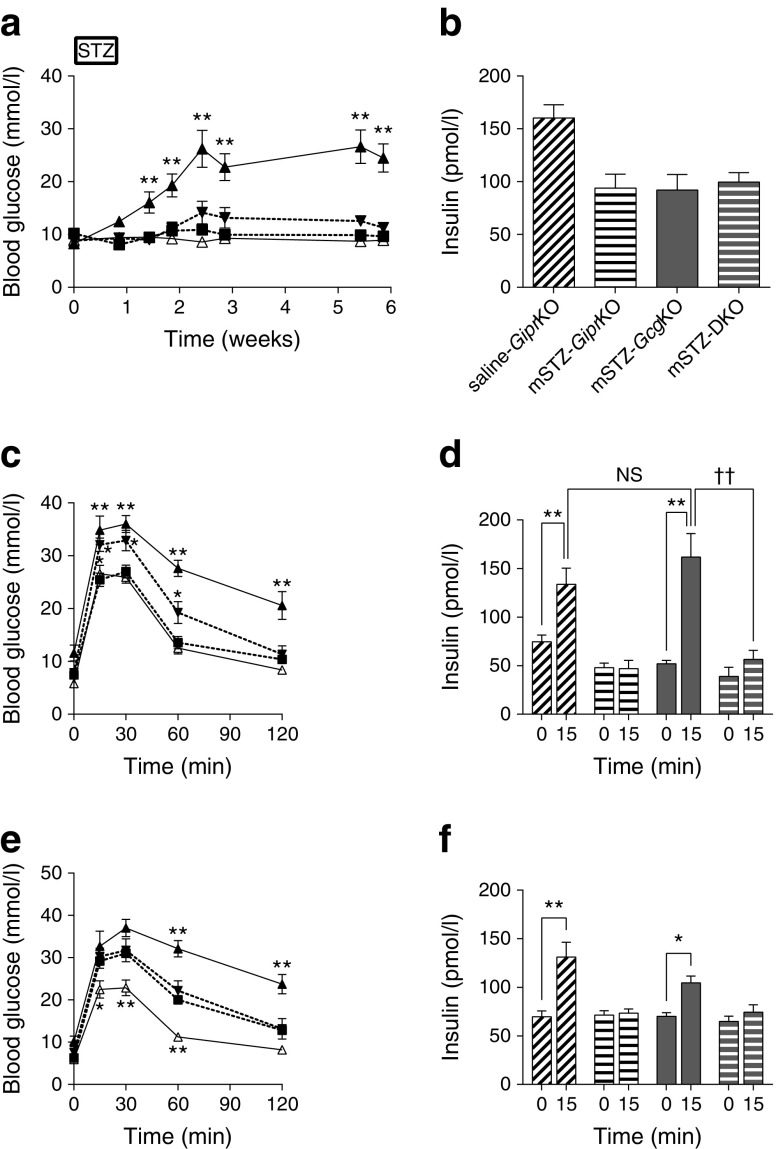
(**a**) Blood glucose levels, (**b**) plasma insulin levels, (**c**, **d**) OGTT and (**e**, **f**) IPGTT in saline-*Gipr*KO, mSTZ-treated *Gipr*KO mice (mSTZ-*Gipr*KO), mSTZ-*Gcg*KO and mSTZ-DKO mice. OGTT and IPGTT were performed 3 and 4 weeks after mSTZ administration, respectively. Plasma insulin levels were evaluated at 5 weeks (**b**). White triangles and solid line, saline-*Gipr*KO; black triangles and solid line, mSTZ-*Gipr*KO; black squares and dashed line, mSTZ-*Gcg*KO; black triangles and dashed line, mSTZ-DKO (**a**, **c**, **e**). Diagonal-striped bars, saline-*Gipr*KO; horizontal-striped bars, mSTZ-GiprKO; dark grey bars, mSTZ-*Gcg*KO; horizontal-striped dark grey bars, mSTZ-DKO (**b**, **d**, **f**). *n* = 8–11, saline-*Gipr*KO; *n* = 6–8, mSTZ-*Gipr*KO; *n* = 5, mSTZ-*Gcg*KO; *n* = 5–6, mSTZ-DKO. *p < 0.05, **p < 0.01 vs mSTZ-treated *Gcg*KO mice (**a**, **c**, **e**). *p < 0.05, **p < 0.01, ^††^ *p* < 0.01; NS, not significant (**d**, **f**)

To assess the role of GIP in glucose homeostasis of mSTZ-treated *Gcg*KO mice, we treated the DKO mice with mSTZ (mSTZ-DKO) and analysed glucose tolerance. Glucose tolerance of DKO mice without STZ treatment was comparable with that of control mice [16]. Blood glucose levels under ad libitum-fed conditions in mSTZ-DKO mice were not significantly different from those in mSTZ-*Gcg*KO mice or non-diabetic *Gipr*KO mice; and plasma insulin levels under ad libitum-fed conditions in mSTZ-DKO mice were not significantly different from those in mSTZ-*Gcg*KO mice (Fig. 3a, b). However, glucose tolerance and insulin secretion during OGTT in mSTZ-DKO mice were impaired compared with those in mSTZ-*Gcg*KO and saline-treated mice (Fig. 3c, d and data not shown), indicating a significant role of GIP in beta cell function under mSTZ-induced damage. On the other hand, no significant difference in glucose levels was observed between mSTZ-*Gcg*KO and mSTZ-DKO mice during IPGTT (Fig. 3e). Significant increase in insulin level in response to i.p. glucose load was observed in mSTZ-*Gcg*KO but not in mSTZ-DKO mice (Fig. 3f). The apparently differential insulin sensitivity observed between these two models might be due to presence or absence of the GIP receptor. Previous studies have shown that mice deficient in GIP action are more sensitive to insulin than control mice [22, 23].

### DPP4i enhances glucose-induced insulin secretion only in mSTZ-treated *Gcg*KO mice

**Fig. 4:**
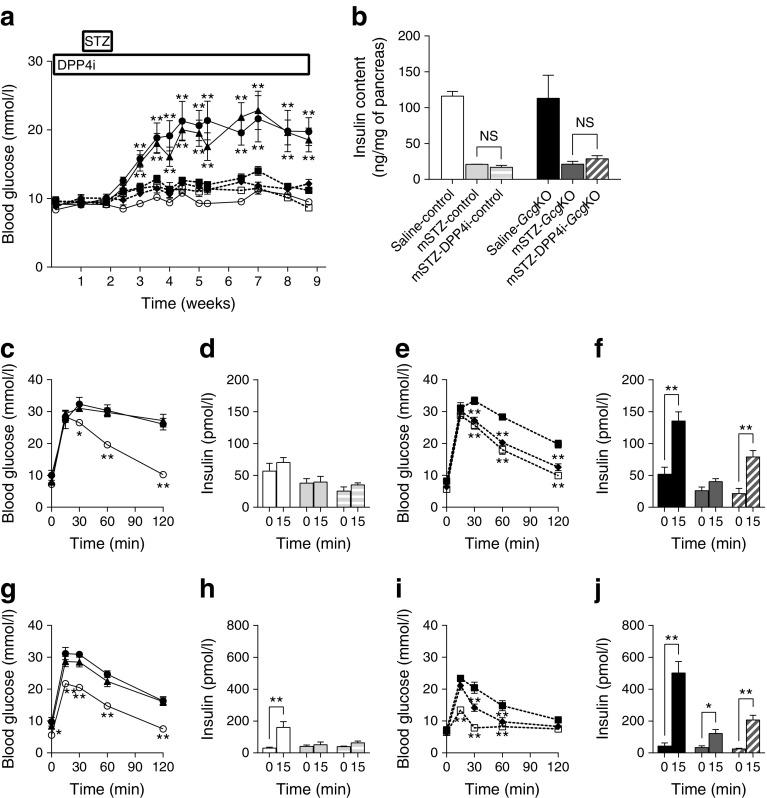
(**a**) Blood glucose levels, (**b**) pancreatic insulin content, (**c**–**f**) IPGTT and (**g**–**j**) OGTT in *Gcg*KO and control mice treated with DPP4i. IPGTT and OGTT were performed at 6 and 7 weeks after mSTZ injection, respectively. *p < 0.05, **p < 0.01, vs saline-treated (**a**, **c**, **e**, **g**, **i**). *p < 0.05, **p < 0.01; NS, not significant (**b**, **d**, **f**, **h**, **j**). White circles and solid line, saline-control; black circles and solid line, mSTZ-control; black triangles and solid line, mSTZ-DPP4i-control; white squares and dashed line, saline-*Gcg*KO; black squares and dashed line, mSTZ-*Gcg*KO; black diamonds and dashed line, mSTZ-DPP4i-*Gcg*KO (**a**, **c**, **e**, **g**, **i**). White bars, saline-control; light grey bars, mSTZ-control; horizontal-striped light grey bars, mSTZ-DPP4i-control; black bars, saline-*Gcg*KO; dark grey bars, mSTZ-*Gcg*KO; diagonal-striped dark grey bars, mSTZ-DPP4i-*Gcg*KO (**b**, **d**, **f**, **h**, **j**). *n* = 4–7, saline-control; *n* = 3–7, mSTZ-control; *n* = 7–8, mSTZ-DPP4i-control; *n* = 4–9, saline-*Gcg*KO; *n* = 5–9, mSTZ-*Gcg*KO; *n* = 5–6, mSTZ-DPP4i-*Gcg*KO

Because GIP and GLP-1 are rapidly inactivated by DPP4 [12], DPP4 inhibitors are widely used for clinical treatment of diabetes as an insulin secretagogue. In addition, GIP and GLP-1 are considered to play a critical role in glucose-induced insulin secretion from pancreatic beta cells and anti-apoptotic action for beta cell survival mediated by DPP4 inhibition [24, 25]. To investigate whether enhancement of GIP signalling by DPP4i improves glucose tolerance and/or protects beta cells in mSTZ-*Gcg*KO mice, DPP4i was administered as shown in Fig. 4a. This treatment improved glucose tolerance by increasing insulin secretion during OGTT in non-diabetic wild-type mice (ESM Fig. 3a, b), but did not during IPGTT (ESM Fig 3c, d). DPP4i treatment also significantly increased active GIP and GLP-1 levels during OGTT as well as under ad libitum-fed status (ESM Fig 3e–h). Treatment with DPP4i did not affect blood glucose levels under ad libitum-fed states in either control or *Gcg*KO mice after mSTZ treatment (Fig. 4a). Pancreatic insulin content was decreased to 18.1 ± 0.2 (%) in control mice and to 18.8 ± 3.4 (%) in *Gcg*KO mice 9 weeks after mSTZ treatment, and DPP4i did not increase pancreatic insulin content in either mSTZ-control mice or mSTZ-*Gcg*KO mice (Fig. 4b). On IPGTT and OGTT, both mSTZ-control and mSTZ-*Gcg*KO mice showed impaired glucose intolerance relative to the corresponding saline-treated animals (Fig. 4c, e, g, i). However, treatment with DPP4i showed differential effects on mSTZ-control and mSTZ-*Gcg*KO mice. Treatment by DPP4i failed to improve glucose intolerance and insulin secretory response in mSTZ-control mice during IPGTT (Fig. 4c, d) and OGTT (Fig. 4g, h). On the other hand, with the same treatment in *Gcg*KO mice, blood glucose levels were significantly reduced at 30, 60 and 120 min during IPGTT (Fig. 4e) and at 30 and 60 min during OGTT (Fig. 4i). In addition, glucose-induced insulin secretion was increased during IPGTT and OGTT by chronic DPP4 inhibition in mSTZ-*Gcg*KO mice (Fig. 4f, j). DPP4i treatment did not improve glucose tolerance or insulin secretion in mSTZ-DKO mice (ESM Fig. 4). These results indicate that insulin secretion by GIP plays an essential role in glucose metabolism in mSTZ-*Gcg*KO mice under treatment with DPP4i. Pancreatic GIP contents were not reduced in hSTZ- or mSTZ-control mice. On the other hand, pancreatic GIP contents were significantly reduced in hSTZ-*Gcg*KO mice but not in mSTZ-*Gcg*KO mice (ESM Fig. 5).

### GIP in *Gcg*KO mice did not enhance beta cell survival after mSTZ treatment

**Fig. 5:**
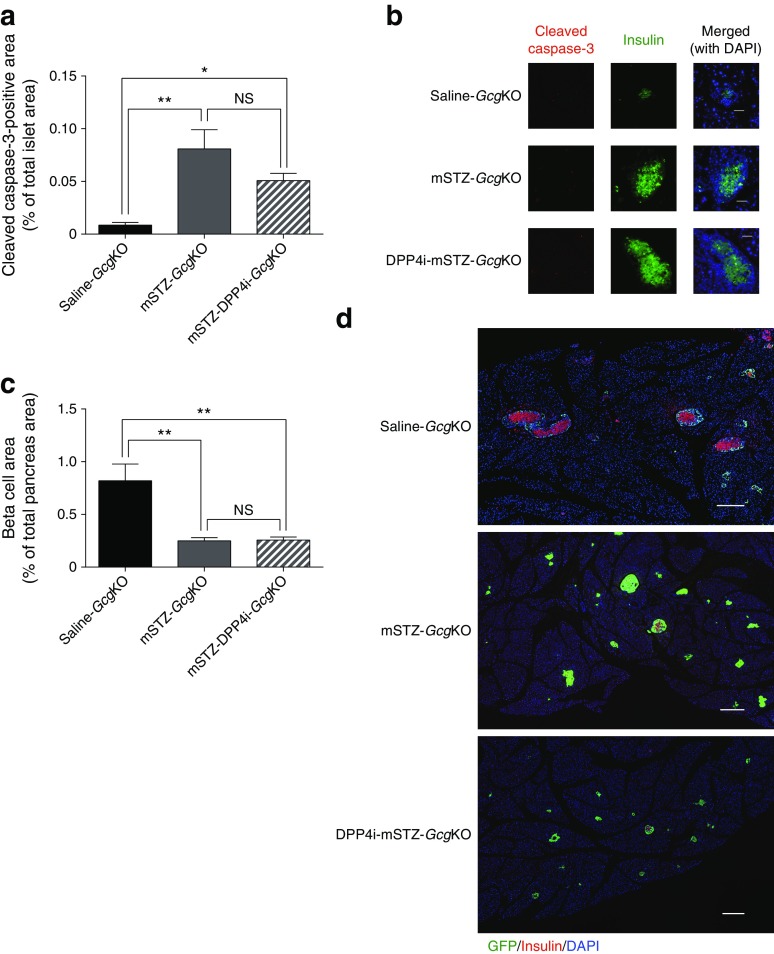
(**a**, **b**) Cleaved caspase-3 immunopositivity and (**c**, **d**) beta cell area of islets from *Gcg*KO mice. (**b**) Representative images of cleaved caspase-3-positive islets. Red, cleaved caspase-3; green, insulin; blue, DAPI. Scale bars, 50 μm. (**d**) Representative images of pancreas. Red, insulin; green, GFP (glucagon); blue, DAPI. Scale bars, 300 μm. Black bars, saline-*Gcg*KO; dark grey bars, mSTZ-*Gcg*KO; diagonal-striped dark grey bars, mSTZ-DPP4i-*Gcg*KO (**a**, **c**). *p < 0.05, **p < 0.01; NS, not significant (*p* = 0.07 for [**a**])

It was reported that endogenous GIP plays a limited role in beta cell survival in STZ-induced diabetes models [25, 26]. To investigate the role of GIP in beta cell survival in the absence of PGDPs including GLP-1, we analysed apoptosis in islets and beta cell mass in the pancreas of mSTZ-*Gcg*KO mice. The percentage of beta cells positive for cleaved caspase-3 was increased significantly by mSTZ treatment. Treatment of mSTZ-*Gcg*KO with DPP4i did not significantly reduce caspase-positive cells (Fig. 5a, b) and beta cell mass in DPP4i-mSTZ-*Gcg*KO mice was comparable with that in mSTZ-*Gcg*KO mice (Fig. 5c, d). Pancreatic insulin content was similar between *Gcg*KO mice and DKO mice 5 weeks after mSTZ treatment (ESM Fig. 6). These results suggest that improvement of insulin secretion and glucose tolerance in mSTZ-*Gcg*KO mice by DPP4i-treatment is mediated through mechanisms other than promotion of beta cell survival.

## Discussion

Glucagon increases hepatic glucose production; insulin inhibits glucose production [27]. The relative contribution of dysregulated glucagon secretion and impairment of insulin secretion to hyperglycaemia in diabetic patients has been a matter of debate [28]. It has been shown recently that administration of STZ to *Gcgr*^−/−^ mice disrupted 90% of the beta cells and abolished glucose-induced insulin secretion yet failed to cause hyperglycaemia [10]. Based on this observation, it has been proposed that hyperglucagonaemia itself plays an essential role in increasing blood glucose levels.

However, in the present study we observed that *Gcg*KO mice lacking both glucagon and GLP-1 developed hyperglycaemia upon hSTZ-induced beta cell ablation. This difference between *Gcgr*^−/−^ and *Gcg*KO is most likely due to the presence or absence of extra-pancreatic GLP-1 action. Several studies have shown that GLP-1 exhibits extra-pancreatic action in increasing insulin sensitivity and modulating glucose metabolism [9, 20, 29]. This is supported by studies employing *Gcgr*^−/−^*Glp1r*^−/−^ double knockout mice and mice with diphtheria toxin mediated-ablation of alpha and L cells (Gluc-DTR). Both models are deficient in GLP-1 action and exhibit hyperglycaemia on STZ treatment, underscoring the critical importance of GLP-1 on glycaemic control under STZ-induced beta cell ablation [30–32]. Nevertheless, there are unique characteristics among *Gcgr*^−/−^*Glp1r*^−/−^, Gluc-DTR and *Gcg*KO mice. *Gcg*KO mice lack all proglucagon-derived peptides throughout life, while there is a decrease in PGDPs in Gluc-DTR mice: GLP-1 synthesis in Gluc-DTR mice returns to normal levels 7 days after injection of diphtheria toxin [33]. GLP-2 is present in *Gcgr*^−/−^*Glp1r*^−/−^ mice, but is reduced or absent in Gluc-DTR and *Gcg*KO mice, respectively. Thus, the presence or absence of GLP-2 and residual glucagon in Gluc-DTR mice does not seem to affect glycaemic control under beta cell ablation.

Nevertheless, *Gcg*KO mice, which lack GLP-1, maintain normoglycaemia after mSTZ treatment, which causes persistent hyperglycaemia in the control mice. Thus, the requirement for insulin to maintain normal blood glucose levels is lower in both *Gcg*KO and *Gcgr*^−/−^ mice [9, 15, 34, 35]. In the present study, plasma insulin levels under ad libitum-fed states were comparable between mSTZ-control and mSTZ-*Gcg*KO mice (Fig. 2b). Insulin levels in *Gcg*KO mice before STZ treatment were not significantly different from those after mSTZ treatment. These findings indicate that insulin plays a critical role in the maintenance of glucose levels in mSTZ-*Gcg*KO mice. Incretins regulate insulin secretion and GIP is the major incretin in *Gcg*KO mice, which lack GLP-1 [36].

Several reports suggest that GIP potentiates the early phase of glucose-induced insulin secretion to contribute to improved glycaemic control [16, 37–39]. GIP also has been reported to contribute to beta cell survival in vitro [40, 41], while GIP overexpression was found to enhance the increment of insulin content induced by high-fat diet feeding by decreasing beta cell apoptosis [39]. We previously reported that GIP was expressed not only in the gastrointestinal tract but also in pancreatic beta cells in *Gcg*KO mice and that GIP hypersecretion contributes to the enhanced glucose-induced insulin secretion and improved glucose tolerance under non-diabetic states in *Gcg*KO mice [16]. These findings led us to investigate whether GIP contributes to resistance to developing diabetes in mSTZ-*Gcg*KO mice.

Moderate beta cell damage abolished insulin secretion in DKO but not in *Gcg*KO mice (Fig. 3). Treatment with DPP4i potentiated glucose-induced insulin secretion and ameliorated glucose intolerance in mSTZ-*Gcg*KO but not in mSTZ-DKO mice (Fig. 4, ESM Fig. 4). These results indicate that GIP played an important role in protecting mice deficient in PDGPs from diabetes. However, treatment with DPP4i did not significantly reduce the number of apoptotic cells in islets (Fig. 5), and blocking GIP actions did not modify pancreatic insulin content in mSTZ-treated mice (ESM Fig. 6). These results indicate that GIP does not contribute to beta cell protection in mSTZ-*Gcg*KO mice but that it does contribute to increase insulin secretion from each of the beta cells.

It was reported recently that GIP and GLP-1 are secreted not only from enteroendocrine K- and L cells but also from pancreatic islets [16, 42–45]. In the present study, pancreatic GIP content in control mice decreased neither by hSTZ treatment nor by mSTZ treatment, most likely because GIP is expressed in pancreatic alpha cells [16, 26, 45]. On the other hand, GIP content in *Gcg*KO pancreas was decreased by hSTZ treatment, confirming our previous results showing that GIP is expressed in beta cells in *Gcg*KO. However, pancreatic GIP content was not changed by mSTZ treatment (ESM Fig. 5). The mechanism underlying the lack of change in pancreatic GIP content under the mSTZ-induced beta cell damage, including that of regeneration of GIP-positive cells, remains to be elucidated.

Islet-derived GLP-1 has been shown to enhance glucose-induced insulin secretion in vitro by using DPP4i in non-diabetic human and mouse islets [46]. In the present study, IPGTT and OGTT analyses suggested that both islet-derived GIP and gut-derived GIP contributes to improving glucose metabolism in mSTZ-*Gcg*KO mice. In, addition, our results indicate that islet-derived GIP can exert an insulinotropic effect even when islet insulin contents are decreased under glucagon-deficient states. We therefore propose that combination therapy with glucagon antagonist and DPP4i might be considered as a therapeutic option to treat diabetes.

## Electronic supplementary material

Below is the link to the electronic supplementary material.

ESM Figures (PDF 961 kb)

## Abbreviations

BW: Body weight
DKO: *Gcg*–*Gipr* double knockout
DPP4: Dipeptidyl peptidase IV
DPP4i: DPP4 inhibitor
*Gcg*KO: Glucagon gene knockout mice, which lack all the PGDPs including glucagon and GLP-1
GIP: Glucose-dependent insulinotropic polypeptide
*Gipr*KO: GIP receptor knockout
GLP-1: Glucagon-like peptide-1
Gluc-DTR: Mice with diphtheria toxin mediated-ablation of alpha and L cells
hSTZ: High-dose streptozotocin
IPGTT: Intraperitoneal glucose tolerance test
mSTZ: Moderate-dose streptozotocin
PGDPs: Proglucagon-derived peptides
STZ: Streptozotocin
