# Supplementary material for: Endogenous GIP ameliorates impairment of insulin secretion in proglucagon-deficient mice under moderate beta cell damage induced by streptozotocin
Source: Diabetologia. 2016 Apr 6;59:1533–41. doi: 10.1007/s00125-016-3935-2 (PMC4901104; doi:10.1007/s00125-016-3935-2)
Supplement: Supplementary file 1 — (PDF 961 kb) [file 125_2016_3935_MOESM1_ESM.pdf]

Electronic Supplemental Material

ESM Fig. 1

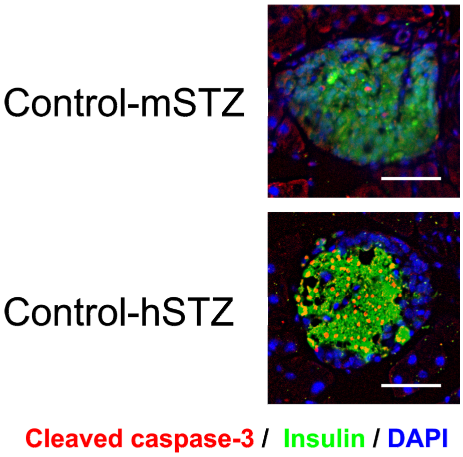

Cleaved caspase-3 expression in islets of hSTZ- and mSTZ-treated control mice.

Fluorescent images of islets from mSTZ-treated and hSTZ-treated control mice. Cleaved caspase-3, red; insulin, green; DAPI, blue. Scale bars, 50 μm.

ESM Fig. 2

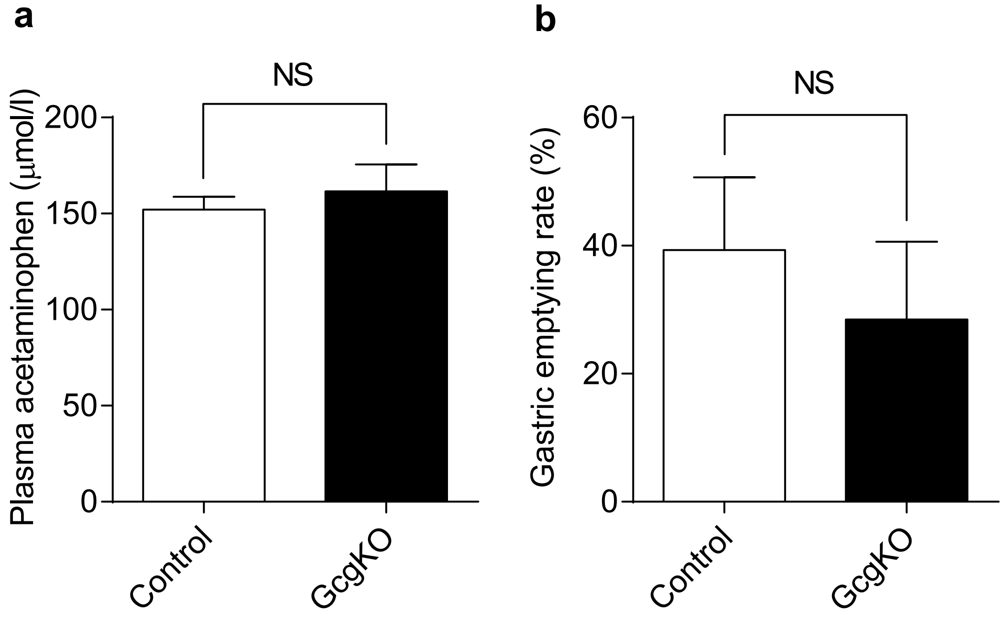

*Liquid (**a**) and solid (**b**) phase of gastric emptying in control and GcgKO mice.*

Acetaminophen absorption test for assessing liquid-phase gastric emptying and evaluation of gastric emptying rate for assessing solid-phase gastric emptying were carried out as previously described [Reference: Ali S, Lamont BJ, Charron MJ, et al. (2011) Dual elimination of the glucagon and GLP-1 receptors in mice reveals plasticity in the incretin axis. J Clin Invest 121:1917–1929]. (**a**) Distilled water containing 100 mg/kg BW (662 μmol/kg) acetaminophen was given orally after overnight (16 hours) fast. Plasma acetaminophen was measured at 15 minutes in control and *Gcg*KO mice. (**b**) Mice were given a standard chow for two hours after overnight (16 hours) fast. NS, not significant. White bars, control mice; black bars, *Gcg*KO mice. *n*=4 per group.

ESM Fig. 3

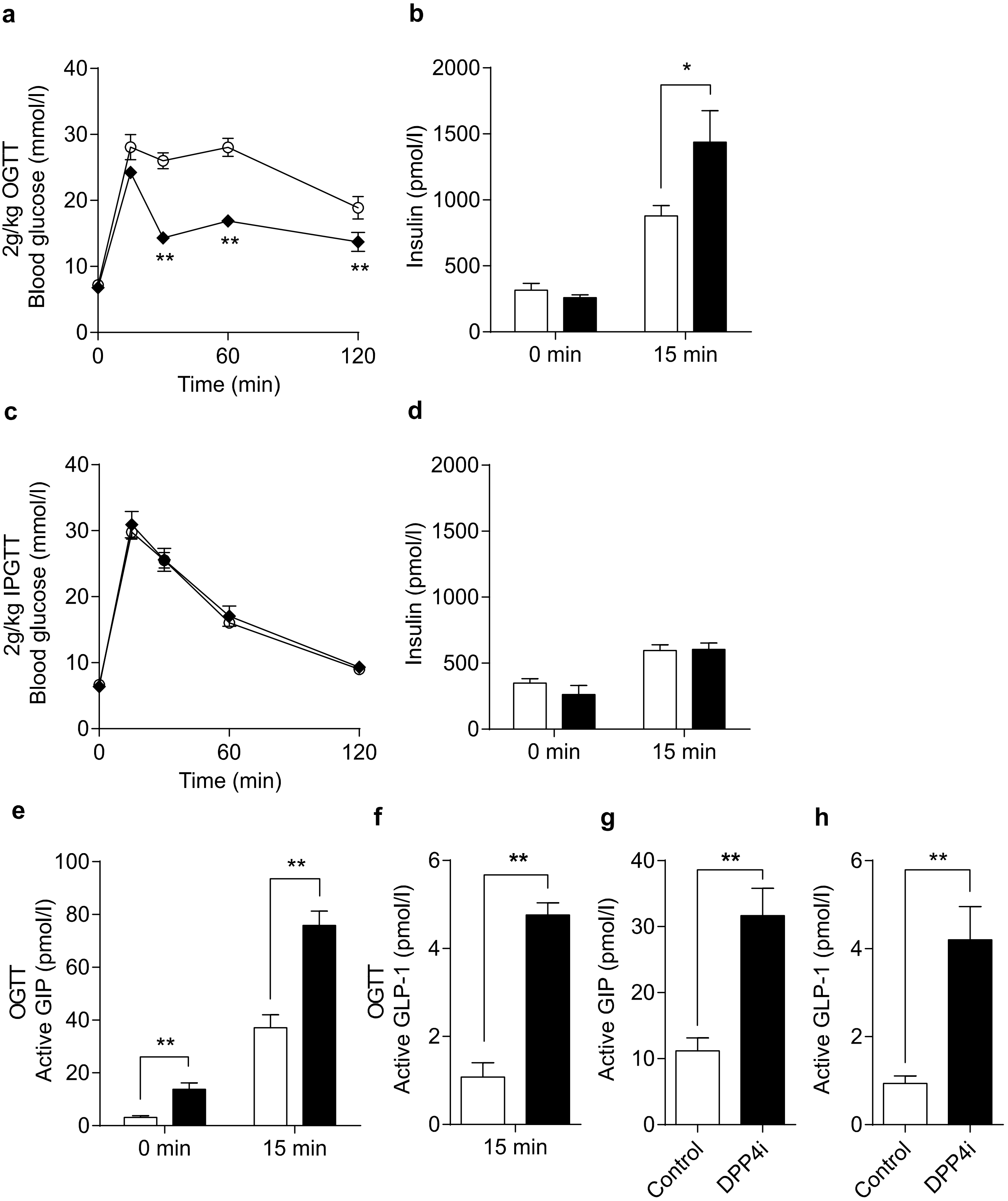

Active GIP and GLP-1 levels during IPGTT, and OGTT in DPP4 inhibitor-treated wild type mice

DPP4 inhibitor treatment was performed in the same dose as mSTZ-DPP4i mice in Figure 4.

OGTT (**a**, **b**) and IPGTT (**c**, **d**) were performed at 4 and 6 weeks, respectively. 2g/kg BW glucose was administered orally or intraperitoneally after 16-hour fast. Plasma active GIP and GLP-1 levels were measured at 0 and/or 15 minutes (**e**, **f**). Plasma active GIP and GLP-1 levels in mice fed *ad libitum* condition (**g**, **h**). White circles and solid line, non-treated wild type mice (control); black diamonds and solid line, DPP4 inhibitor-treated wild type mice (DPP4i). White bars, control; black bars, DPP4i. *n*=6–7 per group.

ESM Fig. 4

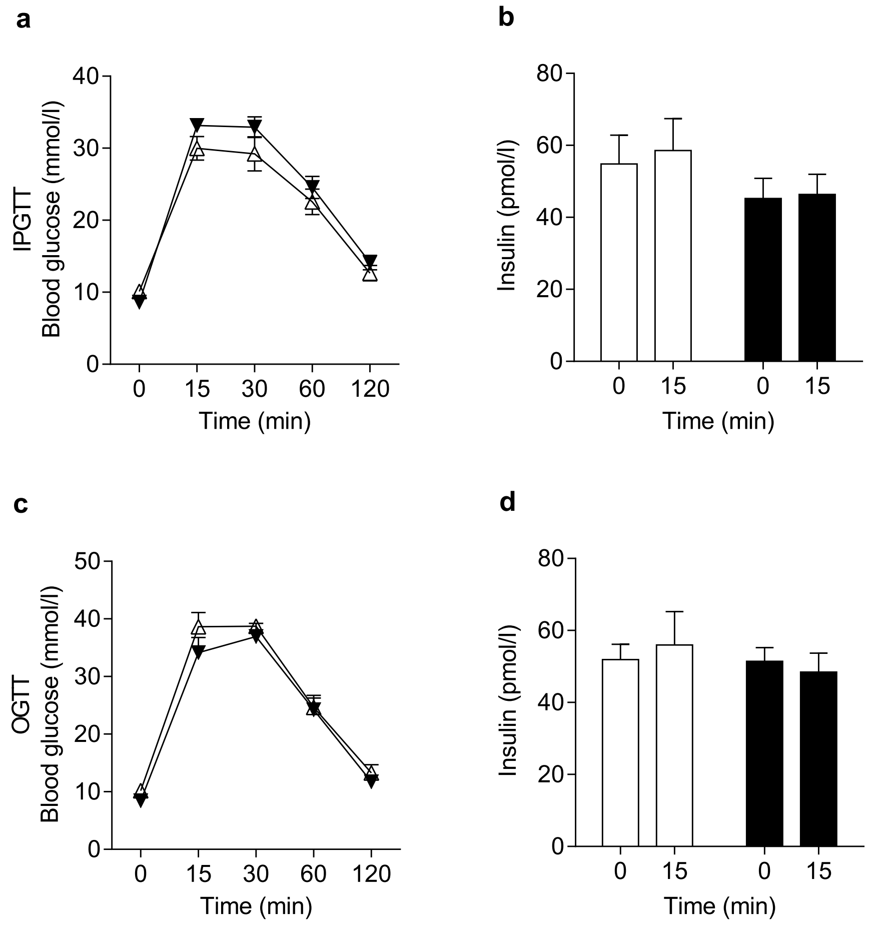

Intraperitoneal and oral glucose tolerance test in DPP4 inhibitor and/or mSTZ-treated DKO mice

DPP4 inhibitor was administered from one week before mSTZ injection throughout the experiment. 2g/kg BW glucose was administered intraperitoneally or orally after 16-hour fast. (**a**, **b**) IPGTT and (**c**, **d**) OGTT were performed at 6 and 8 weeks after mSTZ treatment, respectively. Plasma insulin was measured at 0 and 15 minutes (**b**, **d**). White triangles and solid line, mSTZ-treated DKO mice (mSTZ-DKO); black triangles and solid line, DPP4 inhibitor-treated mSTZ-DKO mice (mSTZ-DPP4i-DKO). White bars, mSTZ-DKO mice; black bars, mSTZ-DPP4i-DKO mice. *n*=5–6, mSTZ-DKO; *n*=6, mSTZ-DPP4i-DKO.

ESM Fig. 5

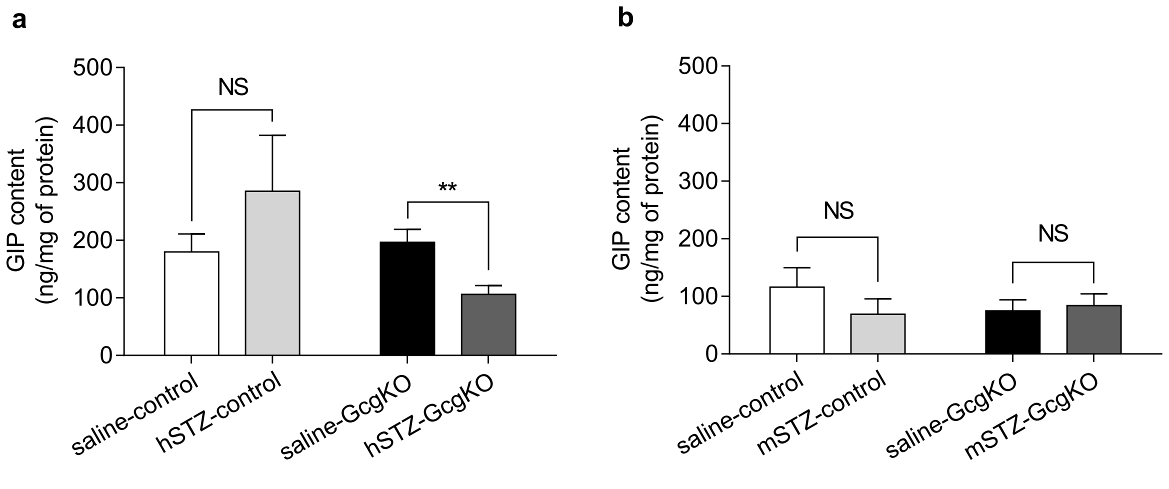

Pancreatic GIP content in control and GcgKO mice after saline, hSTZ, or mSTZ treatment

Pancreatic GIP content was analysed at 9 days after hSTZ treatment (**a**) or at 9 weeks after mSTZ treatment (**b**). White bars, saline-control; light grey bars, hSTZ- or mSTZ-control; black bars, saline-*Gcg*KO; dark grey bars, hSTZ- or mSTZ-*Gcg*KO. $^{**}p<0.01$; NS, not significant. (**a**) n=8, saline-control; n=8, hSTZ-control; n=6, saline-*Gcg*KO; n=6, hSTZ-*Gcg*KO. (**b**) n=5, saline-control; n=6, mSTZ-control; n=5, saline-*Gcg*KO; n=6, mSTZ-*Gcg*KO.

ESM Fig. 6

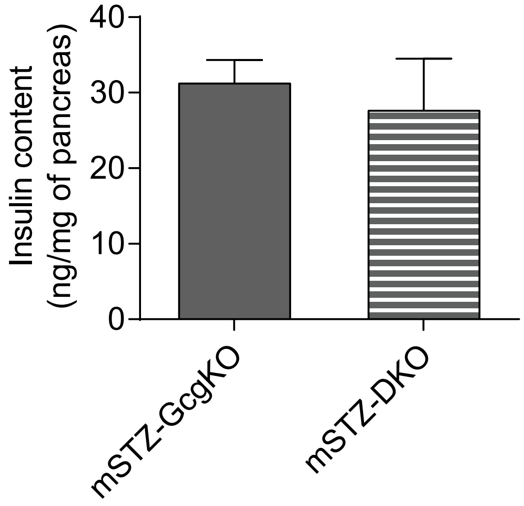

Pancreatic insulin content in GcgKO and DKO mice.

Pancreatic insulin content in *Gcg*KO and DKO mice was analysed at 5 weeks after moderate-dose STZ treatment. Dark grey bars, mSTZ-*Gcg*KO; horizontal-striped dark grey bars, mSTZ-DKO. *n*=5, mSTZ-*Gcg*KO; *n*=6, mSTZ-DKO.
